# Weekly versus triweekly cisplatin-based concurrent chemoradiotherapy for nasopharyngeal carcinoma: a systematic review and pooled analysis

**DOI:** 10.7150/jca.62188

**Published:** 2021-08-28

**Authors:** Jie Tang, Guo-Rong Zou, Xiu-Wen Li, Zhen Su, Xiao-Long Cao, Bi-Cheng Wang

**Affiliations:** 1 Department of Oncology, Panyu Central Hospital, Cancer Institute of Panyu, Guangzhou 511400, China.; 2 Department of Cardiology, Panyu Central Hospital, Guangzhou 511400, China.; 3 Cancer Center, Union Hospital, Tongji Medical College, Huazhong University of Science and Technology, Wuhan 430022, China.

**Keywords:** weekly, triweekly, cisplatin, concurrent chemotherapy, nasopharyngeal carcinoma

## Abstract

**Background:** Weekly and triweekly cisplatin-based concurrent chemoradiotherapy (CCRT) have been used in the treatment of nasopharyngeal carcinoma (NPC).

**Objective:** This study aimed to compare the benefits and risks between the two treatments.

**Methods:** We systematically searched electronic databases for prospective and retrospective clinical studies of NPC patients who received weekly compared with triweekly cisplatin-based CCRT. The primary endpoints comprised overall, failure-free, distant metastasis-free, and locoregional recurrence-free survivals (OS, FFS, DMFS, and LRFS). Secondary endpoints were toxicities.

**Results:** Six studies were included in the systematic review, of which four with 1515 NPC patients were eligible for further pooled analysis. There were no significant differences between weekly and triweekly groups in terms of 5-year OS (odds ratio [OR] 0.95, 95% confidence interval [CI] 0.51-1.79), FFS (OR 1.09, 95% CI 0.67-1.76), DMFS (OR 1.25, 95% CI 0.54-2.92), and LRFS (OR 0.83, 95% CI 0.55-1.25). For grade ≥ 3 toxicities, the weekly group had higher risks of anemia (risk ratio [RR] 2.96, 95% CI 1.12-7.81) and thrombocytopenia (RR 2.75, 95% CI 1.54-4.90), but a lower incidence of vomiting (RR 0.34, 95% CI 0.18-0.63) versus the triweekly group.

**Conclusion and Relevance:** Both weekly and triweekly schedules could be recommended to NPC patients during CCRT. Additionally, hematologic adverse events in weekly strategy and non-hematologic adverse events in triweekly strategy are of higher concern.

## Introduction

In head and neck cancer (HNC), cisplatin-based concurrent chemotherapy (CCRT) is the standard of care. Previously reported meta-analyses showed that the effects of weekly cisplatin CCRT were comparable to triweekly strategy in treating head and neck cancers 1, 2. Subsequently, a randomized phase III trial indicated that triweekly cisplatin was superior to weekly cisplatin in improving locoregional recurrence-free survival (LRFS) 3. Nevertheless, nasopharyngeal carcinoma (NPC) was excluded in these studies.

For patients with locoregionally advanced NPC, the mainstay of therapy is cisplatin-based CCRT as well. However, the 5-year overall survival rates vary from 67.9% to 80.2% when NPC patients receive weekly or triweekly cisplatin-based CCRT 4-7. After reviewing the latest National Comprehensive Cancer Network (NCCN) guidelines on NPC, whether weekly or triweekly administration of cisplatin is recommended has not been clearly clarified 8. According to the cited clinical trials, both strategies are reasonable treatments 9-11.

There is no solid evidence demonstrating the comparable survival outcomes of these two treatments for NPC. Consequently, we conducted this study to synthesize the published results in order to systematically review and pool-analyze the benefits and risks between weekly and triweekly cisplatin-based CCRT in NPC.

## Methods

We conducted this study following the Preferred Reporting Items for Systematic Reviews and Meta-analyses (PRISMA) guideline 12.

### Search strategy and study selection

**Figure 1:**
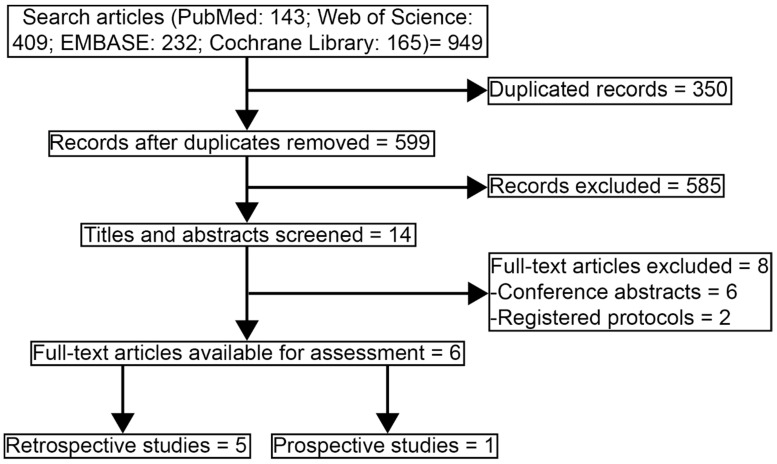
Study selecting process.

The systemic search was done in PubMed, Web of Science, EMBASE, and Cochrane Library using the terms “nasopharyngeal”, “carcinoma or cancer or tumor”, “week or weekly”, “three weeks or 3 weeks or 3-weekly or 3 weekly or three weekly or three-weekly or triweekly”, “cisplatin”, and “trial or study”. The references of relevant published studies were manually searched for further eligible studies. The search was completed up to Dec 13, 2020. Subsequently, the searching process was conducted again on Jun 15, 2021 for updating the newly published records. Moreover, we expanded the literature search to PubMed Central from inception to Jun 15, 2021, which might carry the largest collection of free-full text medical articles among existing databases, and 3443 records were found. Since these records could not be uploaded to the EndNote software, two authors (Jie Tang and Bi-Cheng Wang) screened them carefully, but no more articles were additionally added to this study. Thus, records in PubMed Central database were not displayed in **Figure 1**.

Studies eligible for inclusion met all of the following criteria: (1) previously untreated and non-distant metastatic patients with NPC, (2) participants were treated with weekly cisplatin-based CCRT versus triweekly cisplatin-based CCRT, (3) prospective or retrospective studies were eligible, (4) data of survival outcomes and safety profiles were available, (5) published language was English. Conference abstracts were excluded. Any discrepancies were resolved by discussion.

### Quality assessment

Since only retrospective studies were included in the meta-analysis, the nine-star Newcastle-Ottawa Scale was used to evaluate the quality 13. Scores 7-9 points were defined as high quality, while scores ≤ 6 were as low quality.

### Data extraction

Basic characteristics concerning first author's name, publication year, study design, number of patients, cisplatin dosage, radiotherapy technology, induction or adjuvant chemotherapy regimens were collected. Data of primary endpoints (OS, failure-free survival [FFS, defined as the time from the date of randomization to the date of locoregional failure, distant failure, or death from any cause, whichever occurred first], distant metastasis-free [DMFS, defined as the time from documented distant metastasis or death from any cause], and LRFS [defined as the time from documented locoregional recurrence or death from any cause]) and secondary endpoints (safety profiles) were collected.

### Statistical analysis

All analyses were done using STATA 14.0 software. *p* < 0.05 was considered statistically significant. The survival data were assessed by odds ratios (ORs) and 95% confidence interval (CI). Alternatively, the safety data were evaluated by risk ratios (RRs) and 95% CI. Heterogeneity among the studies was calculated by using the χ^2^ tests. We also quantified the heterogeneity of the results using *I*^2^ statistic percentages. A fixed-effects model (Mantel-Haenszel method) was applied if heterogeneity test showed no statistical significance (*I*^2^ ≤ 50% or *p* ≥ 0.10). Otherwise, a random-effects model was adopted.

## Results

### Search results

**Figure 1** showed the study selection process of articles that were eligible in the systematic review and pooled analysis according to PRISMA 12. 949 records were collected for the initial evaluation. 350 duplicating records were excluded. After reviewing the titles and abstracts, we excluded 585 irrelevant topics. There were 14 studies remaining for further review. Eight studies were excluded as the publication types were conference abstracts (n = 6) or registered protocols (n = 2). Finally, six studies were selected in the systematic review 14-19. Since one study lack survival data and one study was a prospective trial, the other four retrospective studies were eligible in the further pooled analysis, with high qualities assessed by Newcastle-Ottawa scale 16-19.

Basic characteristics of the eligible studies included in the systematic review and pooled analysis were showed in **Table 1**. One study was prospective phase II clinical trials and five were retrospective studies. 3-dimensional conformal radiotherapy (3D-CRT) had been used in two studies and intensity-modulated radiotherapy (IMRT) had been applied in all six studies. In weekly group and triweekly group, respectively, patients were treated with 30-40 mg/m^2^ and 80-100 mg/m^2^. Patients in two studies received cisplatin plus 5-fluorouracil adjuvant chemotherapy, but these patients were not included in the meta-analysis. All patients did not receive any induction chemotherapy.

### Survival outcomes

Survival data were available from four studies with 1515 patients (weekly: 481; triweekly: 1034). Forest plots showed that weekly cisplatin-based CCRT failed to significantly prolong the 5-year OS (OR 0.95, 95% CI 0.51-1.79, *p* = 0.885), FFS (OR 1.09, 95% CI 0.67-1.76, *p* = 0.735), DMFS (OR 1.25, 95% CI 0.54-2.92, *p* = 0.598), and LRFS (OR 0.83, 95% CI 0.55-1.25, *p* = 0.366).

The 5-year rates of OS, FFS, DMFS, and LRFS in weekly group were, respectively, 88.4% (95% CI 81.6-95.3) versus 90.1% (95% CI 88.3-91.9), 83.2% (95% CI 76.5-89.9) versus 82.1% (95% CI 75.4-88.8), 92.0% (95% CI 86.8-97.2) versus 91.1% (95% CI 87.5-94.6), and 93.1% (95% CI 89.7-96.6) versus 93.6% (95% CI 91.5-95.7) compared to triweekly group (**Table 2**).

### Grade ≥ 3 toxicities

For grade ≥ 3 hematologic toxicities, weekly cisplatin-based CCRT significantly increased the risks of anemia (RR 2.96, 95% CI 1.12-7.81, *p* = 0.001), but not thrombocytopenia (RR 1.24, 95% CI 0.96-1.61, *p* = 0.096) and leucopenia (RR 2.75, 95% CI 1.54-4.90, *p* = 0.096) compared to triweekly cisplatin-based CCRT. In terms of grade ≥ 3 non-hematologic toxicities, weekly strategy had a lower risk of vomiting (RR 0.34, 95% CI 0.18-0.63, *p* = 0.001) and similar incidences of nausea (RR 0.55, 95% CI 0.17-1.79, *p* = 0.318) and mucositis (RR 0.81, 95% CI 0.55-1.20, *p* = 0.287) versus triweekly strategy.

## Discussion

In the treatment for NPC patients, it is unclear how to administer CCRT based on the current NCCN guidelines. According to this study, we found that no significant differences were shown in all 5-year survival outcomes between weekly and triweekly treatments. The only prospective phase II study provided similar 3-year results 14. However, weekly regimen was associated with improved quality of life 3 weeks after treatment completion 14.

Since the efficacy of both dosing frequencies are similar, it is also important to consider the dosing of weekly or triweekly cisplatin.

### Weekly 30 mg/m^2^ versus 40 mg/m^2^ cisplatin

In our previous study 20, we found that most prospective randomized clinical trials utilized cisplatin 40 mg/m^2^ weekly. In patients treated with 40 mg/m^2^ cisplatin based CCRT, the 3-year OS rates ranged from 71% to 92% and the 3-year FFS ranged from 57% to 67% 21-23. Hong showed that NPC patients received 30 mg/m^2^ cisplatin had a 5-year OS rate of 68% and a 5-year FFS rate of 50% 4. Based on these data, we cannot directly conclude whether 30 mg/m^2^ or 40 mg/m^2^ has more favorable outcomes for NPC. After careful reading the details, we noticed that the compliance to concurrent cisplatin was a key obstacle because of nausea, vomiting, or hematological toxicities. In Fountzilas's study, no more than 75% of enrolled NPC patients received a cumulative dose of over 200 mg/m^2^ cisplatin 21. 16% of patients in the CCRT alone group in Tan's research had cisplatin discontinued eventually. In Frikha's study, only 22% patients in the CCRT group could receive the full dose of concurrent cisplatin 23. However, for NPC patients in Hong's clinical trial, there were 233 of 240 patients completing the 30 mg/m^2^ cisplatin-based CCRT, with greatly higher treatment compliance than 40 mg/m^2^ cisplatin 4. Based on current clinical practice, we suggest that 30 mg/m^2^ cisplatin with at least six cycles could be a feasible concurrent strategy due to a better tolerability over 40 mg/m^2^/week.

Triweekly 80 mg/m^2^ versus 100 mg/m^2^ cisplatin: In Zhang's study, 98% of patients completed at least two cycles of 100 mg/m^2^ cisplatin-based CCRT 9. The 3-year OS and FFS rates in CCRT group were 85% and 74%. Sun and colleagues reported a 99% rate of patients given CCRT alone in finishing at least two cycles of 100 mg/m^2^ cisplatin during CCRT, with 82% (3-year OS), 77% (5-year OS), 70% (3-year FFS), and 63% (5-year FFS) of survival rates 5, 24. Among patients treated with 80 mg/m^2^ cisplatin-based CCRT alone in Cao's study, 96.8% of participants completed at least two cycles of concomitant cisplatin chemotherapy 6, 25. The 3-year rates of OS and FFS were 88% and 74%, while the 5-year rates were 77% and 63%. Accordingly, both 80 mg/m^2^ and 100 mg/m^2^ cisplatin-based CCRT have highly treatment compliances. There were no obvious differences in survival responses between the two groups. Therefore, it is possible that a cumulative dose of 160 mg/m^2^ cisplatin may be sufficient for CCRT, compared to 200 mg/m^2^.

In clinical practice, if total dose of cisplatin during CCRT is set to 200 mg/m^2^, triweekly 100 mg/m^2^ cisplatin might be a better choice. Alternatively, if the total dose is 160 mg/m^2^, we consider that triweekly 80 mg/m^2^ with two cycles is reasonable. However, the mainly reasons for discontinuation were adverse events.

### Toxicities

We choose six grade ≥ 3 adverse events in analyzing the risks, including anemia, leucopenia, thrombocytopenia, nausea, vomiting, and mucositis, because the data of these toxicities were available from the four studies 16-19. Although we noticed that there were significant differences in anemia and vomiting, the incidences were low and these adverse events could be well managed. Nevertheless, the eligible studies were retrospective researches and did not fully provide the safety profiles. Actually, NPC patients should receive adequate symptomatic supportive care during CCRT to ensure the continuation of cisplatin-based treatments.

Another issue is whether or not concurrent cisplatin schedules will affect survival and toxicity in already chemotherapy-loaded patients, compared to chemotherapy naïve patients. For locoregionally advanced NPC patients, mounts of large-scale prospective clinical trials have demonstrated that induction chemotherapy followed by CCRT is the standard of care 4, 6, 9, 21, 26-30. In a previously published analysis, seven trials were enrolled, with four trials adopting weekly cisplatin schedule and the other three trials adopting triweekly cisplatin schedule 20. Additionally, CCRT plus adjuvant chemotherapy could also be a therapeutic strategy for NPC patients 7, 31. In these studies, both weekly and triweekly strategies were used. However, there is still a lack of head-to-head clinical trials to certify the impacts of different cisplatin-based CCRT schedules on survival outcomes and toxicities in patients who are prescribed to receive induction or adjuvant chemotherapy. Based on our results, both treatments were feasible, which might shed some light and provide possible direction to designing future clinical trials.

## Limitations

There were several limitations that might increase the risk of bias of this meta-analysis. (1) The four eligible studies in pooled analysis were retrospective clinical researches; (2) the weekly cisplatin schedule included 30 mg/m^2^ and 40 mg/m^2^ and the triweekly cisplatin schedule comprised 80 mg/m^2^ and 100 mg/m^2^; (3) only three types of hematologic toxicities (grade ≥ 3 anemia, leucopenia, and thrombocytopenia) and three types of non-hematologic toxicities (grade ≥ 3 nausea, vomiting, and mucositis) were analyzed. In addition, data of nausea and vomiting in Tao's and Wang' studies could not be clearly separated.

## Conclusion and Relevance

Either weekly or triweekly cisplatin during CCRT could be an option for the treatment of NPC. More prospective clinical trials are warranted to confirm our results.

## Figures and Tables

**Figure 2:**
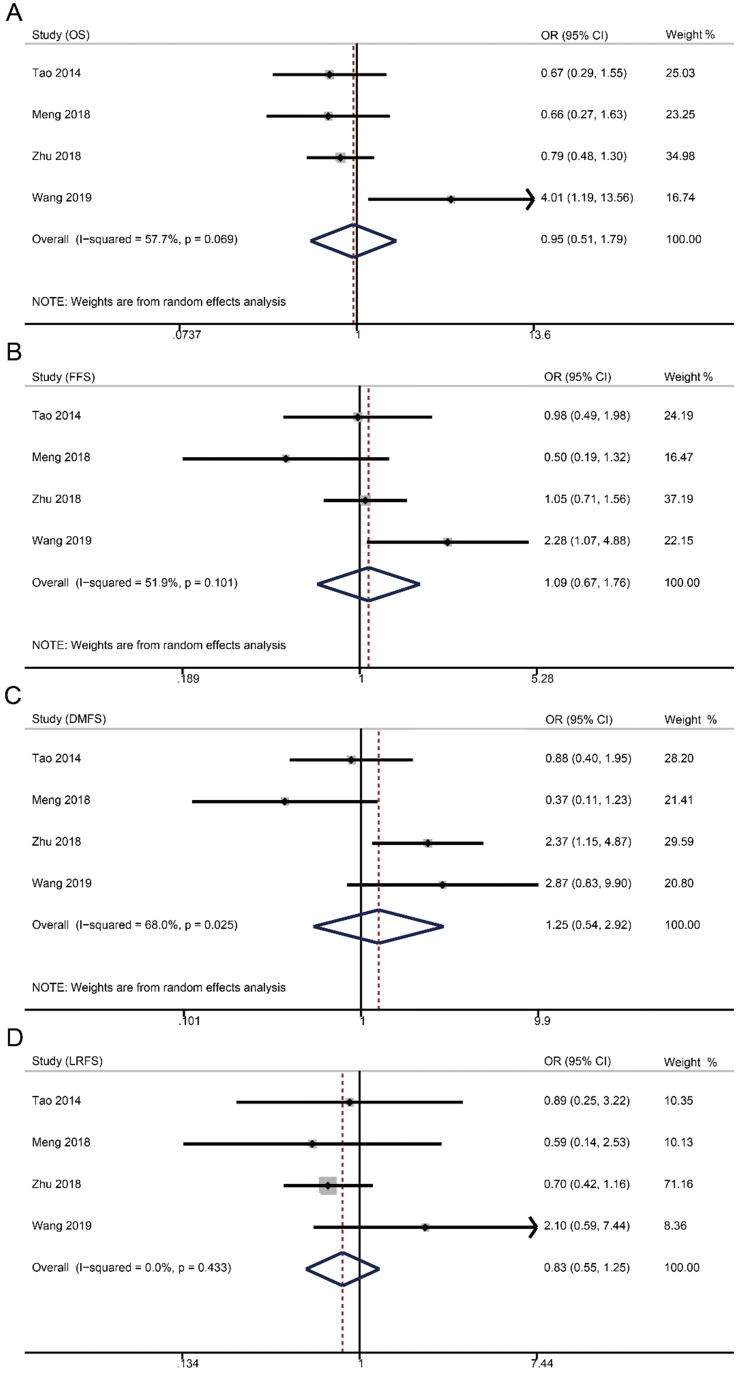
Forest plots of 5-year survival outcomes for weekly versus triweekly cisplatin-based CCRT. (A) overall survival; (B) failure-free survival; (C) distant metastasis-free survival; (D) locoregional recurrence-free survival. OR, odds ratio.

**Figure 3:**
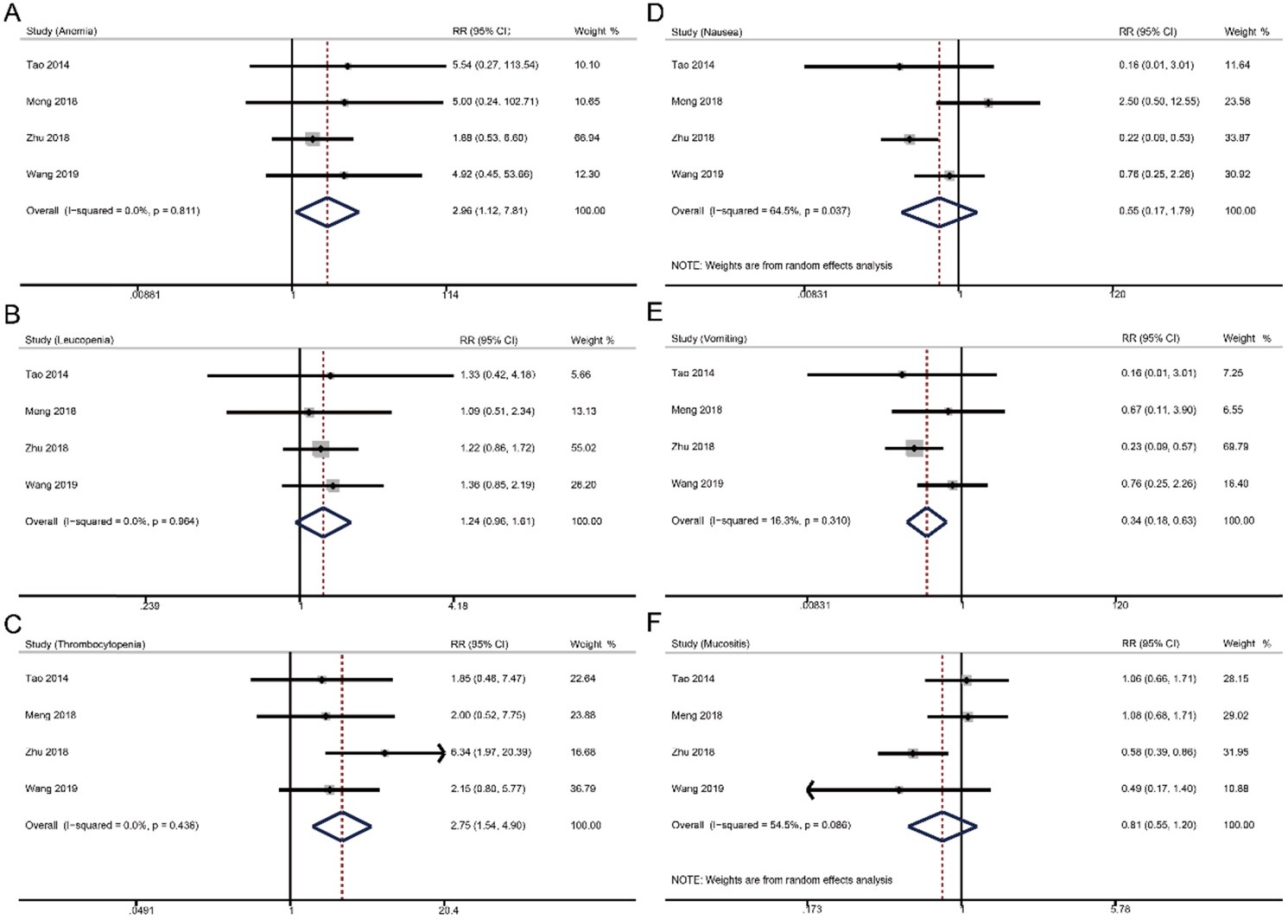
Forest plots of toxicities in comparing the weekly and triweekly cisplatin strategies during CCRT. (A) anemia; (B) leucopenia; (C) thrombocytopenia; (D) nausea; (E) vomiting; (F) mucositis. RR, risk ratio.

**Table 1:** Basic characteristics of the selected studies in the systemic review

Study	Year	Design	Groups	No. patients	Stage	AJCC/UICC	Dosage	Radiotherapy	Induction chemotherapy	Adjuvant chemotherapy	NOS scores
Lee	2016	Prospective	WeeklyTrweekly	5356	II-IVb	5th	40 mg/m^2^100 mg/m^2^	3D-CRT/IMRT	/	Cisplatin and 5-fluorouracil	/
Jagdis	2014	Retrospective	WeeklyTrweekly	4528	II-IVb	7th	40 mg/m^2^100 mg/m^2^	3D-CRT/IMRT	/	Cisplatin and 5-fluorouracil	/
Tao	2014	Retrospective	WeeklyTrweekly	7381	II-IVb	7th	40 mg/m^2^80 mg/m^2^	IMRT	/	/	9
Meng	2018	Retrospective	WeeklyTrweekly	9090	III-IVb	7th	30-40 mg/m^2^80 mg/m^2^	IMRT	/	/	9
Zhu	2018	Retrospective	WeeklyTrweekly	225634	III-IVb	7th	40 mg/m^2^100 mg/m^2^	IMRT	/	/	9
Wang	2019	Retrospective	WeeklyTrweekly	93229	I-IVa	8th	30-40 mg/m^2^80-100 mg/m^2^	IMRT	/	/	9

Abbreviations: AJCC, American Joint Commission on Cancer; UICC, Union for International Cancer Control; 3D-CRT, 3-dimensional conformal radiotherapy; IMRT, intensity-modulated radiotherapy; NOS, Newcastle-Ottawa scale.

**Table 2:** Survival outcomes in the two groups

5-year responses	Rate%, 95% CI	
	weekly	triweekly
OS	88.4, 81.6-95.3	90.1, 88.3-91.9
FFS	83.2, 76.5-89.9	82.1, 75.4-88.8
DMFS	92.0, 86.8-97.2	91.1, 87.5-94.6
LRFS	93.1, 89.7-96.6	93.6, 91.5-95.7

Abbreviations: OS, overall survival; FFS, failure-free survival; DMFS: distant metastasis-free survival; LRFS: locoregional recurrence-free survival; 95% CI, 95% confidence interval.
